# Supplementary material for: Shifting employment and perceptions of household responsibilities during early stages of the COVID-19 pandemic in Nevada, USA
Source: PLoS One. 2024 Nov 11;19(11):e0309906. doi: 10.1371/journal.pone.0309906 (PMC11554153; doi:10.1371/journal.pone.0309906)
Supplement: S1 File — (DOCX) [file pone.0309906.s001.docx]

**Appendix Table 1a. Frequencies and proportions in covariates by employment status change from a subsample (n=535) of Nevada adults in December 2020 who reported working full or part-time before the pandemic. The p-value was computed by the chi-square test.**

|  | **Has not changed** | | **Reduced hours** | | **Fired or laid off** | | **I quit working voluntarily** | | **Working more hours** | |  |
| --- | --- | --- | --- | --- | --- | --- | --- | --- | --- | --- | --- |
|  | **(N=262 ; %=49.43)** | | **(N=72 ; %=13.58)** | | **(N=108 ; %=20.38)** | | **(N=47 ; %=8.87)** | | **(N=41 ; %=7.74 )** | |  |
| **Variable** | **N** | **%** | **N** | **%** | **N** | **%** | **N** | **%** | **N** | **%** | **P-value** |
| Gender (Missing = 10) |  |  |  |  |  |  |  |  |  |  | 0.1060 |
| Female | 118 | 45.21 | 39 | 14.94 | 55 | 21.07 | 31 | 11.88 | 18 | 6.9 |  |
| Male | 139 | 52.65 | 33 | 12.5 | 53 | 20.08 | 16 | 6.06 | 23 | 8.71 |  |
| Race & Ethnicity (Missing = 20) |  |  |  |  |  |  |  |  |  |  | <0.0001 |
| Non-Hispanic White | 164 | 53.42 | 34 | 11.07 | 58 | 18.89 | 33 | 10.75 | 18 | 5.86 |  |
| Hispanic, Latino(a), or Spanish | 34 | 41.46 | 15 | 18.29 | 18 | 21.95 | 4 | 4.88 | 11 | 13.41 |  |
| Non-Hispanic Black or African American | 19 | 38.78 | 10 | 20.41 | 14 | 28.57 | 3 | 6.12 | 3 | 6.12 |  |
| Non-Hispanic Asian | 11 | 55 | 2 | 10 | 2 | 10 | 2 | 10 | 3 | 15 |  |
| Multiple or other races | 26 | 45.61 | 8 | 14.04 | 14 | 24.56 | 4 | 7.02 | 5 | 8.77 |  |
| Age (Missing = 20) |  |  |  |  |  |  |  |  |  |  | <0.0001 |
| 18-29 | 37 | 38.95 | 11 | 11.58 | 23 | 24.21 | 9 | 9.47 | 15 | 15.79 |  |
| 30-44 | 52 | 50.98 | 17 | 16.67 | 26 | 25.49 | 3 | 2.94 | 4 | 3.92 |  |
| 45-64 | 116 | 52.97 | 26 | 11.87 | 45 | 20.55 | 14 | 6.39 | 18 | 8.22 |  |
| 65+ | 48 | 48.48 | 16 | 16.16 | 12 | 12.12 | 21 | 21.21 | 2 | 2.02 |  |
| Education level (Missing = 11) |  |  |  |  |  |  |  |  |  |  | 0.0214 |
| Less than high school diploma | 5 | 41.67 | 3 | 25 | 2 | 16.67 | 1 | 8.33 | 1 | 8.33 |  |
| Grade 12 or GED (high school graduate) | 48 | 40.34 | 22 | 18.49 | 33 | 27.73 | 8 | 6.72 | 8 | 6.72 |  |
| College 1 year to 3 years (some college or technical school) | 83 | 45.36 | 25 | 13.66 | 42 | 22.95 | 18 | 9.84 | 15 | 8.2 |  |
| College 4 years or more (college graduate) | 60 | 52.17 | 10 | 8.7 | 24 | 20.87 | 11 | 9.57 | 10 | 8.7 |  |
| Graduate or professional degree | 64 | 67.37 | 10 | 10.53 | 6 | 6.32 | 8 | 8.42 | 7 | 7.37 |  |
| Income level (Missing = 81) |  |  |  |  |  |  |  |  |  |  | <0.0001 |
| $0 - $30,000 | 26 | 24.07 | 19 | 17.59 | 43 | 39.81 | 13 | 12.04 | 7 | 6.48 |  |
| $30,001 - $65,000 | 51 | 45.95 | 17 | 15.32 | 26 | 23.42 | 9 | 8.11 | 8 | 7.21 |  |
| $65,001 - $105,000 | 66 | 58.41 | 16 | 14.16 | 14 | 12.39 | 7 | 6.19 | 10 | 8.85 |  |
| $105,001 or more | 85 | 69.67 | 8 | 6.56 | 12 | 9.84 | 11 | 9.02 | 6 | 4.92 |  |
| Residential area (Missing = 5) |  |  |  |  |  |  |  |  |  |  | 0.5236 |
| Urban | 229 | 49.04 | 65 | 13.92 | 99 | 21.2 | 40 | 8.57 | 34 | 7.28 |  |
| Rural | 33 | 52.38 | 7 | 11.11 | 9 | 14.29 | 7 | 11.11 | 7 | 11.11 |  |
| Five samples are missing in employment status change. |  |  |  |  |  |  |  |  |  |  |  |

**Appendix Table 2a. Frequencies and proportions in covariates by housework time change from a subsample (n=535) of Nevada adults in December 2020 who reported working full or part-time before the pandemic. The p-value was computed by the chi-square test.**

|  | **More time** | | **Same time** | | **Less time** | |  |
| --- | --- | --- | --- | --- | --- | --- | --- |
|  | **(N=211; %=39.59)** | | **(N=293; %=54.97)** | | **(N=29; %=5.44)** | |  |
| **Variable** | **N** | **%** | **N** | **%** | **N** | **%** | **P-value** |
| Gender (Missing = 7) |  |  |  |  |  |  | 0.0165 |
| Female | 118 | 44.7 | 128 | 48.48 | 18 | 6.82 |  |
| Male | 93 | 35.23 | 160 | 60.61 | 11 | 4.17 |  |
| Race & Ethnicity (Missing = 17) |  |  |  |  |  |  | 0.0286 |
| Non-Hispanic White | 112 | 36.25 | 183 | 59.22 | 14 | 4.53 |  |
| Hispanic, Latino(a), or Spanish | 39 | 48.15 | 38 | 46.91 | 4 | 4.94 |  |
| Non-Hispanic Black or African American | 25 | 49.02 | 19 | 37.25 | 7 | 13.73 |  |
| Non-Hispanic Asian | 9 | 45 | 11 | 55 | 0 | 0 |  |
| Multiple or other races | 20 | 35.09 | 34 | 59.65 | 3 | 5.26 |  |
| Age (Missing = 17) |  |  |  |  |  |  | 0.0002 |
| 18-29 | 51 | 53.68 | 41 | 43.16 | 3 | 3.16 |  |
| 30-44 | 51 | 49.04 | 44 | 42.31 | 9 | 8.65 |  |
| 45-64 | 75 | 34.4 | 129 | 59.17 | 14 | 6.42 |  |
| 65+ | 28 | 27.72 | 70 | 69.31 | 3 | 2.97 |  |
| Education level (Missing = 7) |  |  |  |  |  |  | 0.9379 |
| Less than high school diploma | 6 | 54.55 | 4 | 36.36 | 1 | 9.09 |  |
| Grade 12 or GED (high school graduate) | 49 | 41.18 | 64 | 53.78 | 6 | 5.04 |  |
| College 1 year to 3 years (some college or technical school) | 74 | 39.57 | 101 | 54.01 | 12 | 6.42 |  |
| College 4 years or more (college graduate) | 43 | 37.07 | 68 | 58.62 | 5 | 4.31 |  |
| Graduate or professional degree | 36 | 37.89 | 54 | 56.84 | 5 | 5.26 |  |
| Income level (Missing = 79) |  |  |  |  |  |  | 0.0213 |
| $0 - $30,000 | 48 | 44.44 | 52 | 48.15 | 8 | 7.41 |  |
| $30,001 - $65,000 | 54 | 48.21 | 51 | 45.54 | 7 | 6.25 |  |
| $65,001 - $105,000 | 31 | 27.68 | 75 | 66.96 | 6 | 5.36 |  |
| $105,001 or more | 47 | 37.9 | 73 | 58.87 | 4 | 3.23 |  |
| Residential area (Missing = 2) |  |  |  |  |  |  | 0.0444 |
| Urban | 195 | 41.49 | 251 | 53.4 | 24 | 5.11 |  |
| Rural | 16 | 25.4 | 42 | 66.67 | 5 | 7.94 |  |
| Two samples are missing in housework time change. |  |  |  |  |  |  |  |

**Appendix Table 3a. Odds ratios of employment status change for covariates from a subsample (n=535) of Nevada adults in December 2020 who reported working full or part-time before the pandemic.**

|  | **Reduced hours vs. Has not changed** | | | **Fired or laid off vs. Has not changed** | | | **I quit working voluntarily vs. Has not changed** | | | **Working more hours vs. Has not changed** | | |  |
| --- | --- | --- | --- | --- | --- | --- | --- | --- | --- | --- | --- | --- | --- |
| **Variable** | **OR** | **95% CI** | | **OR** | **95% CI** | | **OR** | **95% CI** | | **OR** | **95% CI** | | **P-value** |
| Gender |  |  |  |  |  |  |  |  |  |  |  |  | 0.3570 |
| Female | 1.59 | 0.84 | 3.00 | 1.05 | 0.60 | 1.81 | 1.87 | 0.89 | 3.94 | 1.26 | 0.56 | 2.83 |  |
| Male |  | Reference |  |  | Reference |  |  | Reference |  |  | Reference |  |  |
| Race & Ethnicity |  |  |  |  |  |  |  |  |  |  |  |  | 0.8909 |
| Non-Hispanic White |  | Reference |  |  | Reference |  |  | Reference |  |  | Reference |  |  |
| Hispanic, Latino(a), or Spanish | 1.29 | 0.51 | 3.26 | 1.12 | 0.50 | 2.50 | 0.77 | 0.21 | 2.77 | 2.49 | 0.83 | 7.42 |  |
| Non-Hispanic Black or African American | 2.27 | 0.87 | 5.92 | 1.72 | 0.70 | 4.22 | 0.96 | 0.25 | 3.74 | 1.25 | 0.25 | 6.38 |  |
| Non-Hispanic Asian | 1.08 | 0.21 | 5.57 | 0.30 | 0.03 | 2.56 | 1.04 | 0.18 | 5.84 | 1.82 | 0.33 | 9.96 |  |
| Multiple or other races | 0.99 | 0.33 | 2.99 | 1.34 | 0.57 | 3.17 | 0.64 | 0.17 | 2.48 | 1.65 | 0.47 | 5.82 |  |
| Age |  |  |  |  |  |  |  |  |  |  |  |  | 0.0431 |
| 18-29 |  | Reference |  |  | Reference |  |  | Reference |  |  | Reference |  |  |
| 30-44 | 1.41 | 0.51 | 3.87 | 1.22 | 0.52 | 2.87 | 0.30 | 0.07 | 1.30 | 0.35 | 0.10 | 1.27 |  |
| 45-64 | 1.12 | 0.43 | 2.92 | 1.02 | 0.46 | 2.24 | 0.58 | 0.19 | 1.77 | 0.61 | 0.22 | 1.70 |  |
| 65+ | 1.62 | 0.54 | 4.84 | 0.84 | 0.31 | 2.28 | 2.14 | 0.70 | 6.56 | 0.26 | 0.05 | 1.40 |  |
| Education level |  |  |  |  |  |  |  |  |  |  |  |  | 0.9602 |
| Less than high school diploma |  | Reference |  |  | Reference |  |  | Reference |  |  | Reference |  |  |
| Grade 12 or GED (high school graduate) | 1.84 | 0.28 | 12.04 | 1.54 | 0.24 | 9.89 | 2.05 | 0.18 | 23.74 | 1.57 | 0.14 | 17.53 |  |
| College 1 year to 3 years (some college or technical school) | 0.88 | 0.39 | 1.97 | 1.00 | 0.50 | 2.00 | 1.32 | 0.45 | 3.85 | 1.07 | 0.37 | 3.08 |  |
| College 4 years or more (college graduate) | 0.59 | 0.21 | 1.70 | 1.27 | 0.57 | 2.86 | 1.47 | 0.45 | 4.84 | 1.35 | 0.40 | 4.52 |  |
| Graduate or professional degree | 0.74 | 0.27 | 2.08 | 0.42 | 0.14 | 1.22 | 0.94 | 0.26 | 3.43 | 0.83 | 0.20 | 3.41 |  |
| Income level |  |  |  |  |  |  |  |  |  |  |  |  | <0.0001 |
| $0 - $30,000 |  | Reference |  |  | Reference |  |  | Reference |  |  | Reference |  |  |
| $30,001 - $65,000 | 0.38 | 0.16 | 0.92 | 0.24 | 0.11 | 0.50 | 0.29 | 0.10 | 0.82 | 0.46 | 0.14 | 1.51 |  |
| $65,001 - $105,000 | 0.34 | 0.14 | 0.82 | 0.12 | 0.06 | 0.28 | 0.22 | 0.07 | 0.66 | 0.50 | 0.15 | 1.61 |  |
| $105,001 or more | 0.12 | 0.04 | 0.36 | 0.08 | 0.03 | 0.19 | 0.29 | 0.10 | 0.82 | 0.35 | 0.09 | 1.31 |  |
| Residential area |  |  |  |  |  |  |  |  |  |  |  |  | 0.6586 |
| Urban | 1.26 | 0.47 | 3.41 | 1.59 | 0.64 | 3.95 | 0.99 | 0.35 | 2.81 | 0.60 | 0.20 | 1.84 |  |
| Rural |  | Reference |  |  | Reference |  |  | Reference |  |  | Reference |  |  |
|  |  |  |  |  |  |  |  |  |  |  |  |  |  |

**Appendix Table 4a. Demographic distribution of the full sample (n=1000) and regression sample (n=766) of Nevada adults in December 2020.**

|  |  | **N = 766** | | **N = 1000** | |  |
| --- | --- | --- | --- | --- | --- | --- |
| **Variable** | **Level** | **N** | **%** | **N** | **%** |  |
| Gender | Female | 412 | 53.79 | 544 | 54.89 |  |
|  | Male | 354 | 46.21 | 447 | 45.11 |  |
| Race & Ethnicity | Non-Hispanic White | 519 | 67.75 | 670 | 68.93 |  |
|  | Hispanic, Latino(a), or Spanish | 91 | 11.88 | 107 | 11.01 |  |
|  | Non-Hispanic Black or African American | 67 | 8.75 | 79 | 8.13 |  |
|  | Non-Hispanic Asian | 21 | 2.74 | 56 | 5.76 |  |
|  | Multiple or other races | 68 | 8.88 | 60 | 6.17 |  |
| Age | 18-29 | 95 | 12.40 | 114 | 11.75 |  |
|  | 30-44 | 103 | 13.45 | 125 | 12.89 |  |
|  | 45-64 | 244 | 31.85 | 293 | 30.21 |  |
|  | 65+ | 324 | 42.30 | 438 | 45.15 |  |
| Education level | Less than high school diploma | 24 | 3.13 | 33 | 3.34 |  |
|  | Grade 12 or GED (high school graduate) | 169 | 21.67 | 218 | 22.09 |  |
|  | Some College | 286 | 37.34 | 361 | 36.58 |  |
|  | College 4 years or more (college graduate) | 152 | 19.84 | 196 | 19.86 |  |
|  | Graduate or professional degree | 138 | 18.02 | 179 | 18.14 |  |
| Income level | $0-$30,000 | 214 | 27.94 | 229 | 28.00 |  |
|  | $30,001-$65,000 | 214 | 27.94 | 225 | 27.51 |  |
|  | $65,001-$105,000 | 173 | 22.58 | 187 | 22.86 |  |
|  | $105,001 or more | 165 | 21.54 | 177 | 21.64 |  |
| Residential area | Urban | 649 | 84.73 | 849 | 84.90 |  |
|  | Rural | 117 | 15.27 | 151 | 15.10 |  |
|  |  |  |  |  |  |  |

**Appendix Table 5a. Odds ratios of household level housework time change for covariates from a subsample (n=535) of Nevada adults in December 2020 who reported working full or part-time before the pandemic.**

| **Variable** | **OR** | **95% CI** | | **P-value** |
| --- | --- | --- | --- | --- |
| Gender |  |  |  | 0.3908 |
| Female | 0.84 | 0.57 | 1.25 |  |
| Male |  | Reference |  |  |
| Race & Ethnicity |  |  |  | 0.9108 |
| Non-Hispanic White |  | Reference |  |  |
| Hispanic, Latino(a), or Spanish | 1.00 | 0.56 | 1.81 |  |
| Non-Hispanic Black or African American | 0.80 | 0.42 | 1.52 |  |
| Non-Hispanic Asian | 0.83 | 0.30 | 2.32 |  |
| Multiple or other races | 1.19 | 0.62 | 2.28 |  |
| Age |  |  |  | 0.0257 |
| 18-29 |  | Reference |  |  |
| 30-44 | 1.29 | 0.69 | 2.42 |  |
| 45-64 | 2.17 | 1.22 | 3.89 |  |
| 65+ | 2.24 | 1.13 | 4.43 |  |
| Education level |  |  |  | 0.6398 |
| Less than high school diploma |  | Reference |  |  |
| Grade 12 or GED (high school graduate) | 0.32 | 0.07 | 1.52 |  |
| College 1 year to 3 years (some college or technical school) | 0.86 | 0.52 | 1.45 |  |
| College 4 years or more (college graduate) | 0.90 | 0.50 | 1.65 |  |
| Graduate or professional degree | 1.06 | 0.55 | 2.03 |  |
| Income level |  |  |  | 0.1411 |
| $0 - $30,000 |  | Reference |  |  |
| $30,001 - $65,000 | 0.98 | 0.56 | 1.70 |  |
| $65,001 - $105,000 | 1.70 | 0.96 | 3.01 |  |
| $105,001 or more | 0.99 | 0.55 | 1.78 |  |
| Residential area |  |  |  | 0.2763 |
| Urban | 0.71 | 0.39 | 1.31 |  |
| Rural |  | Reference |  |  |
|  |  |  |  |  |

**Appendix Table 6a. Demographics from the subsample of Nevada adults who did more housework (n=323) in December 2020.**

| **Variable** |  | **Sample** | **Sample %** |
| --- | --- | --- | --- |
| Gender (Missing = 0) |  |  |  |
|  | Female | 189 | 58.51 |
|  | Male | 134 | 41.49 |
| Race & Ethnicity |  |  |  |
| (Missing = 10) |  |  |  |
|  | Non-Hispanic White | 189 | 60.38 |
|  | Hispanic, Latino(a), or Spanish | 49 | 15.65 |
|  | Non-Hispanic Black or African American | 32 | 10.22 |
|  | Non-Hispanic Asian | 12 | 3.83 |
|  | Multiple or other races | 31 | 9.90 |
| Age |  |  |  |
| (Missing = 10) |  |  |  |
|  | 18-29 | 58 | 18.53 |
|  | 30-44 | 60 | 19.17 |
|  | 45-64 | 102 | 32.59 |
|  | 65+ | 93 | 29.71 |
| Education level | |  |  |
| (Missing = 5) | |  |  |
|  | Less than high school diploma | 15 | 4.72 |
|  | Grade 12 or GED (high school graduate) | 68 | 21.38 |
|  | College 1 year to 3 years (some college or technical school) | 112 | 35.22 |
|  | College 4 years or more (college graduate) | 67 | 21.07 |
|  | Graduate or professional degree | 56 | 17.61 |
| Income level |  |  |  |
| (Missing = 58) |  |  |  |
|  | $0-$30,000 | 75 | 28.3 |
|  | $30,001-$65,000 | 75 | 28.3 |
|  | $65,001-$105,000 | 53 | 20 |
|  | $105,001 or more | 62 | 23.4 |
| Residential area | |  |  |
| (Missing = 0) | |  |  |
|  | Urban | 291 | 90.1 |
|  | Rural | 32 | 9.91 |
|  |  |  |  |
| Note: Percentages may add up to more than 100 percent due to rounding. Sample percentage excludes missing cases. | | | |
