# Supplementary material for: Shifting employment and perceptions of household responsibilities during early stages of the COVID-19 pandemic in Nevada, USA
Source: PLoS One. 2024 Nov 11;19(11):e0309906. doi: 10.1371/journal.pone.0309906 (PMC11554153; doi:10.1371/journal.pone.0309906)
Supplement: Attachment — Submitted filename: 1.8.24 PLOS ONE Reviewer responses.docx [file pone.0309906.s002.docx]

When submitting your revision, we need you to address these additional requirements.

1. Please ensure that your manuscript meets PLOS ONE's style requirements, including those for file naming. The PLOS ONE style templates can be found at

<https://journals.plos.org/plosone/s/file?id=wjVg/PLOSOne_formatting_sample_main_body.pdf> and

<https://journals.plos.org/plosone/s/file?id=ba62/PLOSOne_formatting_sample_title_authors_affiliations.pdf>.

2.  You indicated that ethical approval was not necessary for your study. We understand that the framework for ethical oversight requirements for studies of this type may differ depending on the setting and we would appreciate some further clarification regarding your research. Could you please provide further details on why your study is exempt from the need for approval and confirmation from your institutional review board or research ethics committee (e.g., in the form of a letter or email correspondence) that ethics review was not necessary for this study? Please include a copy of the correspondence as an ""Other"" file.

To clarify, the research ethics committee did review our survey and research protocol, however, it did not go to the full IRB review board. The UNLV Office of Research Integrity determined that our study met the criteria for “Exempt Review.” Exempt review is defined as follows: “The code of federal regulations (45 CFR 46.101(b)) identifies several categories of minimal risk research as exempt from the Federal Policy for the Protection of Research Subjects. This determination must not be made by the PI, but by the IRB or someone appointed by the IRB. For more information, see the U.S. Health and Human Services website, “[Exempt Research and Research That May Undergo Expedited Review](http://www.hhs.gov/ohrp/policy/hsdc95-02.html)”.

Our study protocol included only minimal risks. We did not ask about overly sensitive issues (such as abuse or sexual activity, for example), nor did we collect any identifying information that might be tracked back to the participants. We have attached the official letter stating the exempt status from the UNLV Office of Research Integrity-Human Subjects.

To enhance clarity, we also modified the methods section to read: “The Office of Research Integrity at [blinded] reviewed the study protocol and survey and determined it to be exempt from full IRB review due to the minimal risk of harm and anonymous nature of the survey (December 3, 2020; protocol number 1688442-2).”

3.  In the ethics statement in the Methods, you have specified that verbal consent was obtained. Please provide additional details regarding how this consent was documented and witnessed, and state whether this was approved by the IRB .

We obtained verbal consent, which was approved by the Office of Research Integrity. We have modified and added the following to the methods section: “All participants were told the purpose of the research study, the length of time the survey would take to complete, and that their participation was voluntary. All participants verbally consented to participating in the survey by agreeing to the following statement: “I agree to participate in this study. I am at least 18 years of age.” All participants completed the survey in English. The Office of Research Integrity at [blinded] reviewed the study protocol and survey and determined it to be exempt from full IRB review due to the minimal risk of harm and anonymous nature of the survey.”

4.  Thank you for stating in your Funding Statement:

“This project was partially supported by the U.S. Centers for Disease Control and Prevention as part of a subaward totaling $3.4 million from the Nevada Division of Public and Behavioral Health's Epidemiology and Laboratory Capacity award. The contents are those of the author(s) and do not necessarily represent the official views of, nor an endorsement by, the U.S. Centers for Disease Control and Prevention or the U.S. government.
The funders had no role in study design, data collection and analysis, decision to publish, or preparation of the manuscript.”

Please provide an amended statement that declares *all* the funding or sources of support (whether external or internal to your organization) received during this study, as detailed online in our guide for authors at <http://journals.plos.org/plosone/s/submit-now>.  Please also include the statement “There was no additional external funding received for this study.” in your updated Funding Statement.

Please include your amended Funding Statement within your cover letter. We will change the online submission form on your behalf.

We added the statement “There was no additional external funding received for this study.” And also added: “Remaining support came from the UNLV School of Public Health.”

Reviewer #1: Thank you for the opportunity to read this very interesting paper. The paper has many strengths that should be of interest to the journal audience. Thus, the following suggestions are around enhancing the presentation for publication and clarifying aspects of the data and reporting.
I will go by line number for the most part. If not, I will try to be as specific as possible in noting the area I am speaking about.

Thank you for taking the time to review and provide feedback on our manuscript. It is enhanced significantly as a result of your feedback.

Overall
The manuscript should be shortened

Thank you for this suggestion. We appreciate the need to shorten the paper to communicate our findings most efficiently. However, we also believe that this paper tackles a complex question that requires contextualization and explanation in order to properly understand. Given this and also the reviewers’ suggestions to provide additional details in a number of areas, it would be difficult to decrease the word count without significantly changing the meaning of the paper and making it less clear.

Abstract
[Line 47] Background:. It is necessary to include the country.

We added USA to the title and the abstract’s background.

Where are values and p-values? (p < .001). Authors must specify it. P values showing the differences between groups should be given.

P-values have been added to the abstract.

Methods. Did you use some scales? If not, it is necessary to detailed the variables

Thank you for pointing this out. It is difficult to add significant detail given the short format of the abstract, but we added the following to the abstract methods section: “Using a cross-sectional telephone survey of 1,000 Nevada adults, this study compared respondent experiences and perceptions of employment and time spent on housework…”

1. Introduction
Authors must speak more about gender, status, types of works, and race, the adverse working conditions related to COVID-19, which are the consequences of shifting employment. What is the meaning of household responsibilities?

Please consider stating clear in your text which was the exact understudied population (population of interest) and how it was defined.

The population of interest is Nevada adults, which is stated in the concluding paragraph and the paper’s main aim (see below comment).

The prevalence and incidence of COVID-19 in the area of study during the period of study should be discussed.

We added the following to the introduction line 74-75: “Nevada had over 39 new COVID-19 hospital admissions and 94 cumulative deaths per 100,000 residents [4].”

It is not clear why the study was necessary

We modified the language in the concluding paragraph. Line 98-102 now reads: “A better understanding of employment changes and household responsibilities among Nevada adults is necessary and especially important because of Nevada’s diverse population mix and economy, which were particularly vulnerable to the COVID-19 pandemic. A better understanding of the challenges created by COVID-19 can help with recovery and mitigation and to plan for future emergencies.”

Authors must finish with the main aim.

Thank you for this suggestion. We have modified the language in the concluding paragraph and moved the sentence containing the main aim to be the final sentence of this paragraph.

2. Materials and Methods
Did the authors calculate the needed sample size? Please, clarify. How was the sample size determined? Did the authors test power calculation? How was the sample chosen? Authors must specify it.

We conducted a power analysis based on chi-square analysis. To detect the small effect size of 0.1 required a sample size ranging from 964-1927. We maximized the sample size given the budget we had. This resulted in us being able to collect a sample of 1000 NV adults. Still, our sample is large enough to detect effect sizes between 0.099 and 0.140, which are still small. Please see the below power analysis tables. We added the following language to the methods section to clarify this point: “A power calculation based on the chi-square test indicates that this sample size of 1,000 can detect effect sizes between 0.099 and 0.140.”

Do the authors have a study protocol? The study protocol should be described in detail.
Which is the ID number? (ID number…..:2020). Interventionary studies involving animals or humans, and other studies require ethical approval must list the authority that provided approval and the corresponding ethical approval code. Please include the date and code register number of ethics committee.

Thank you for bringing this to our attention. Please see comment above about the ethics committee review. Our study and protocol were reviewed and determined to be exempt from *full* review due to the minimal risk of harm and anonymous nature of the survey. We also added the date (Dec 3, 2020) and study protocol number (1688442-2).

Please add the response options for each demographic variable in the study. There isn’t enough detail to repeat the experiments.

We added all options for each demographic variable.

Are any potential confounding factors considered?

All of the confounding factors considered in the study are listed in the methods.

DESIGN AND PROCEDURE: they should specify the design of the study they have carried out, and describe thoroughly how the data collection process was carried out, as well as issues such as voluntariness of participation and/or anonymity.

This study used a cross-sectional design, which is stated in the methods section Line 106. We also added details about the ethical considerations, including voluntariness and anonymity in line 117-120 (see comments above as well).

POPULATION and SAMPLE: It is necessary to describe the population size of the Nevada, and from this data, provide a calculation of the sample size necessary for the results to be meaningful. It is also necessary to specify the inclusion and exclusion criteria for the study sample.

We have added the population of Nevada (n’s) to table 1. We also included language on the range of the effect size based on our power calculation. See the comment above for the whole table. We added the following language to clarify inclusion criteria: “To participate in the study, a respondent had to be: a resident of Nevada, able to be contacted via a phone line, over the age of 18 years, and willing to complete the survey in English.”

VARIABLES: In relation to the items that were created "ad hoc", it is also necessary to better describe how these items were agreed (literature review, expert consensus, etc.).

Thank you for the comment. We have added language briefly indicating how the survey was constructed.

ETHICAL CONSIDERATIONS: You should include a sub-heading under Methods that describes these issues: provide the reference number of the Ethics Committee approval, describe how the confidentiality of the data has been guaranteed.

We included this information in the methods section under *Data collection and survey*.

3. Results
Table 1. Please, provide the n and not only %

We have added the n’s to table 1.

At last, but not least, I recommend you to make available your data in an open repository. I think it will make this scientific process more transparent, and it allows other researchers to replicate your results.

Thank you for the comment. We fully support the idea of making the data available to other researchers.

However, IRB approval was obtained without the provision that the collected data would be made publicly available and protocols for data release were not submitted to or vetted by the IRB.

Furthermore, although we anonymized the data for our internal analysis, given some of the small areas and populations that were included in the study (for example, rural or frontier areas of NV), we want to take all precautions against the possibility that someone could identify an individual respondent based on their answers and defeat the anonymization that was conducted on the dataset if it were placed in a public repository. Upon acceptance to PLOS ONE, we will request our Office of Research Integrity to review the data release policy for approval. Once approved, our data can be provided to researchers upon request from the authors, as stated in our Data Availability Statement and consistent with PLOS One data availability policy.

4. Discussion
I think that the discussion section could be shortened by not repeating survey results

Thank you for the suggestion. The additional discussion of some of the results was necessary to provide context and clarity to the reader. As here we were discussing some of the more granular findings, it was not possible to simply refer to the findings of "Table X" without further explanation.

Moreover, some points were not discussed, i.e., the participants were assessed from December, 2020, since a fatigue scenario could exist due to Covid-19 social restrictions; how could this factor impact these participants?

Thank you for this suggestion. We added the following to the limitations section: “Relatedly, pandemic fatigue and pandemic burnout due to an unprecedented pandemic and associated restrictions may have played a role in participation and general perceptions and behaviors at the time of the survey [55, 56].”

Limitations related with the type of methodology used. Limitations regarding representativeness of respondents should be better addressed. Authors must specify it.

Thank you for this comment. We have addressed this limitation in detail. The manuscript states: “The generalizability of findings may be limited due to demographic differences between the sample population and the state of Nevada. Most notably, compared to the state, the sample population had a much higher proportion of respondents who were aged 65 or older and a much lower proportion of respondents under 45 years; a higher percentage of respondents who were White, female, and held a graduate or professional degree; and a lower percentage of Hispanics, Asians, and those reporting “other” or multiple races. Respondents were limited to Nevada and completed the survey in English, which may limit linguistic, economic, and geographic generalizability.”

The fact of having a convenience sample should be included in the limitations of the study.

Thanks for this recommendation. We added the following to the limitations section line 374: “The sample was one of convenience, so those who responded may differ in some way from those who did not.”
I wish you all the best.

Reviewer #2: Intro: First paragraph- would be helpful to have death estimates at time of survey instead of July 2022

Thank you for this suggestion. We updated this first paragraph with the most recent numbers (Dec 2023), and added incidence (weekly hospital admissions) and prevalence (cumulative deaths) figures for the time of the survey in NV in Dec 2020 in line 74-75 of the intro.

Intro: lines 91-92, it is unclear at time of survey what type of closures were in place for gaming industry

The restrictions varied across 2020 and included closures of businesses and schools, stay-at-home orders, and capacity reductions; people also may have chosen to stay at home because they were fearful of transmission. This likely led to people staying at home much more than before the COVID pandemic. This assumption is also consistent with some findings from the literature (see, for example, Engle, S., Stromme, J., & Zhou, A. (2020). Staying at home: mobility effects of covid-19. Available at SSRN 3565703. http://dx.doi.org/10.2139/ssrn.3565703.)

Methods- more details are needed about the sampling frame (list of phone numbers) and sampling procedures. Who provided this frame? Were quotas employed for age-sex? etc.

The data management company contacted all of the participants from their internal list of potential respondents. “No quotas were employed. We added the following to the methods: Participants were contacted via landline (n=408) and cellphone (n=592) between 5 p.m. and 9 p.m. local time by a market research firm who maintains the contact information for participants.”

Methods: Please provide a statistical justification for the sample size

Thank you for this suggestion. We conducted a power analysis based on chi-square analysis. To detect the small effect size of 0.1 required a sample size ranging from 964-1927. We maximized the sample size given our budget. This resulted in us being able to collect a sample of 1000 NV adults. Still, our sample is large enough to detect effect sizes between 0.099 and 0.140, which are still small enough. Please see the below power analysis tables. We added the following language to the methods section to clarify this point: “A power calculation based on the chi-square test indicates that this sample size of 1,000 can detect effect sizes between 0.099 and 0.140.”

Methods: The response rate is a result and not a method. More details about the disposition codes and survey rates are needed. See AAPOR standard guidelines.

Thank you for pointing this out. We moved the response rate to the results section. We also added information about the power analysis and the effect size our sample was able to detect (see above comment). We limited the change to this given the length of the manuscript and other comments suggesting that we cut down the manuscript so as not to overwhelm readers.

Results: Please be consistent with decimal point reporting (i.e sometimes you report 67%, 67.0% and 67.00%

Thank you for pointing this out. We made all decimal points in the results consistent.

Results: line 185- reword "moderately significant" to something else like, "Although not significant...list pvalue

Thank you, we modified the language in line 174-175

Results: Table 1 shows demographics of full sample (n=1000) but sample size of 777 is used for main analyeses (Table 5). Please include a supplemental table that looks at demographic distribution of the full sample and regression sample to see if there is any potential selection bias.

We added appendix table 4a. This table displays the distribution of each sample.

Results/Methods: The authors mentioned restricting data set to those without missing data. Income level has the largest amount of missing data (n=192). Did the authors look at relaxing this criteria so that a larger dataset could be used for analyses.

Thank you for the question. We did explore the model with and without the income variable. The model examining employment status change (Table 5) with the income variable included has the AIC = 1542.287, which is smaller than the AIC = 1550.152 in the model without the income variable. Therefore, having the income variable included in the model makes the multinomial logistic regression better.

In the model examining housework time change (Table 6), using the binary logistic regression, the AUCs of the ROC in the model with and without the income variable is very close (0.631 vs. 0.630). Thus, removing the income variable will not make a significant difference in the quality of the model.

To maintain consistency, we kept the income variable in all relevant models.

Result Table 4...there is an error in percentages for males (29.85% is in both columns)

Thank you for pointing this out, it has been corrected to 70.15.

Results table 7. I would defer to a proper statistician, but it seems like this could be more robustly assessed through an interaction term. If you are presenting Table 7 as a main finding, please include the demographics of this sub-sample.

Thank you. We have added the demographic breakdown of this subsample to Appendix Table 5a.

Results: why not present weighted estimates for some of the key outcomes? especially given the biases the authors mentioned in the demographics of the sample.

We thank the reviewer for this suggestion. We are confident in our findings without the weighting given that the power analysis indicated the ability to detect rather small effect sizes (0.099 and 0.140).

Abstract- the reporting that women have more household work is misleading. Overall, there was minimal difference in less/more/no diff by gender.

Thank you for raising this. We have modified the abstract conclusion to read: “Results demonstrate a disproportionate burden in employment change and/or more household responsibilities on lower income and younger respondents, and females reporting they are more likely to take on additional housework.”

Limitations: Need to add that study was (probably, I'm guessing) not powered for sub-group analyses

Thank you. We added the following to the limitations section: “The sample size for the subgroup analysis was smaller and power was not calculated.”


Results and Abstract... overall I think the results section could be tightened up a little bit. It seems like there is a lot of mention in covariates that are significant in univariate models but not in multivariate models. Table 5 and 6 are the main adjusted regression tables where only age was significantly associated for both outcomes. Quite surprisingly, gender and race/ethnicity are not significantly associated. This gets a little lost in the presentation of results and the discussion. For example, the paragraph starting at line 342. "Responses also revealed the elevated toll of the pandemic on females when it comes to housework?. Consider tempering the conclusion

Thank you for this suggestion. Univariate analysis did reveal a gender disparity, which lost significance once the covariates were added to the model. We believe that we clearly state this in the referenced paragraph. Further, the subsample analysis confirmed the toll on women, with an increased odds of perceiving themselves as doing more of the work. This gender disparity in housework is supported in other literature as well.
