# Supplementary material for: Shifting employment and perceptions of household responsibilities during early stages of the COVID-19 pandemic in Nevada, USA
Source: PLoS One. 2024 Nov 11;19(11):e0309906. doi: 10.1371/journal.pone.0309906 (PMC11554153; doi:10.1371/journal.pone.0309906)
Supplement: Attachment — Submitted filename: review.docx [file pone.0309906.s003.docx]

Thank you for your interesting research. I think it will be even better when it is analyzed properly!

1. I have several questions about study participants and selection.

1) I understand that the survey was conducted by the firm, but who were the subjects registered with this firm? For example, supermarket users or online shoppers?

2) Nowhere did it say how to select the subject. Normally, if there was a list of contacts, there would be some way to randomly select participants from that list, but I wonder if such a method was used.

3) Is there ever more than one participant from a single household in this survey?　 If more than one person is participating, it is better to make an adjustment for each household. This is because if the husband responds that he spends more time at home and thus does more housework, the wife may respond that she does less housework, and their answers would be influenced by the same household.

4) Why are there so many missings when you are communicating directly by phone?

5) I understand that there is missing in household income, but if the respondent had indicated that he/she did not want to answer the question, you could make a no answer category. It would reduce the percentage of missing. Also, you asked about household income, but it is possible that young people, such as 18 years old, may have answered that they did not know. Was there any trend when looking at those who categorized missing by age?

2. For the chi-squared test, all of the expected counts be at least 5 in each cell. However, several categories have the expected counts was below 5, for example, the expected counts of non-Hispanic Asians who responded "Reduced hour" in the Race and Ethnicity category was less than 5. The analysis method is not appropriate. Authors should calculate the p-value using the fisher exact test, etc. If you have a large number of categories, some with very small expected numbers, alternatively, you should consider to summarize categories that are few in number for chi square test.

3. The author responded that “Univariate analysis did reveal a gender disparity, which lost significance once the covariates were added to the model.”

As for the analysis method, you say it is the univariate model, but I think it will be an unadjusted model. It is not adjusted for confounding at all, so it would be better to check it in several ways. For example, if there is a difference in the association with outcomes by gender, you could introduce an interaction term into the model, and if that shows a significant difference, you could stratify by gender and look at the association with the other covariate.

The same can be done for age. Since there are many age groups, you can add one covariate at a time to the same model to see which variable attenuates the odds ratio, and thereby see the effect of the variable. It is believed that presenting such a method would further lead to better discussions and conclusions
