# Supplementary material for: Shifting employment and perceptions of household responsibilities during early stages of the COVID-19 pandemic in Nevada, USA
Source: PLoS One. 2024 Nov 11;19(11):e0309906. doi: 10.1371/journal.pone.0309906 (PMC11554153; doi:10.1371/journal.pone.0309906)
Supplement: Attachment — Submitted filename: Response to reviewr comments. 7.1.24.docx [file pone.0309906.s004.docx]

1) I understand that the survey was conducted by the firm, but who were the subjects registered with this firm? For example, supermarket users or online shoppers?

Response: Thank you for your inquiry. For cell phone data, the survey firm utilized the Telcordia Terminating Point Master (TPM) data, which is a proprietary database. This database includes detailed information about telephone number blocks within the North American Numbering Plan (NANP).

To clarify, the TPM data is not a list of survey subjects such as supermarket users or online shoppers. Instead, it is a technical resource that telecommunications companies, regulatory bodies, and other related entities use for managing and operating telephone networks. The data includes records for area codes and exchanges, broken down into 1000-number and 100-number blocks.

In the context of our survey, the TPM data was used to ensure comprehensive and representative coverage of telephone numbers within Nevada. This methodology helps in achieving a broad and unbiased sample of telephone users across different geographic regions, rather than targeting specific types of consumers like supermarket users or online shoppers.

For landlines – the firm utilized Directory-Listed Landline telephone samples.

The following has been added to the results: data collection and survey section: Cellphone lines came from a proprietary data source with comprehensive coverage in Nevada and landlines from directory-listed numbers. Of these sources, phone numbers from individuals residing in Nevada were randomly selected and dialed.

2) Nowhere did it say how to select the subject. Normally, if there was a list of contacts, there would be some way to randomly select participants from that list, but I wonder if such a method was used.

Response: From the participant information from cellphone and landlines listed above, participants that were located in Nevada were randomly selected and dialed by the survey firm.

3) Is there ever more than one participant from a single household in this survey?　 If more than one person is participating, it is better to make an adjustment for each household. This is because if the husband responds that he spends more time at home and thus does more housework, the wife may respond that she does less housework, and their answers would be influenced by the same household.

Response: No, only one participant per household participated in the survey. We added this to the results section.

4) Why are there so many missings when you are communicating directly by phone?

Response: The “missing” is a combination of “don’t know” and the participant choosing not to answer the question, as participants had the right to choose not to answer. However, only 32% of the “missing” data are the “don’t know” response.

5) I understand that there is missing in household income, but if the respondent had indicated that he/she did not want to answer the question, you could make a no answer category. It would reduce the percentage of missing. Also, you asked about household income, but it is possible that young people, such as 18 years old, may have answered that they did not know. Was there any trend when looking at those who categorized missing by age?

Response: We thank the reviewer for their feedback. The majority of the missing data are responses where participants refused to answer. Upon examining the "don't know" responses by age, we found that only 10 individuals (22%) in the younger age category responded this way. Additionally, when analyzing all income categories within this age group, no apparent trend was observed.

2. For the chi-squared test, all of the expected counts be at least 5 in each cell. However, several categories have the expected counts was below 5, for example, the expected counts of non-Hispanic Asians who responded "Reduced hour" in the Race and Ethnicity category was less than 5. The analysis method is not appropriate. Authors should calculate the p-value using the fisher exact test, etc. If you have a large number of categories, some with very small expected numbers, alternatively, you should consider to summarize categories that are few in number for chi square test.

Response: We thank the reviewer for this suggestion. Cell frequencies less than 5 do not violate the assumption of the chi-square test, rather “expected frequencies” should not be less than 5. Cell frequencies less than 5 may have an expected frequency larger than 5, depending on corresponding marginal frequencies and the total frequency. The only variable that violated this assumption is the cross-table between race and employment status change in Table 2. Because Fisher's exact test does not work in such a 5 by 5 table, we computed a simulated p-value in Fisher's exact test in R, resulting in a p-value of 0.0005. The simulation is based on the following paper: [An Efficient Method of Generating Random R × C Tables with Given Row and Column Totals - Patefield - 1981 - Journal of the Royal Statistical Society: Series C (Applied Statistics) - Wiley Online Library](https://rss.onlinelibrary.wiley.com/doi/10.2307/2346669)

We updated the p-value in table 2. This does not change overall findings.

3. The author responded that “Univariate analysis did reveal a gender disparity, which lost significance once the covariates were added to the model.”

As for the analysis method, you say it is the univariate model, but I think it will be an unadjusted model. It is not adjusted for confounding at all, so it would be better to check it in several ways. For example, if there is a difference in the association with outcomes by gender, you could introduce an interaction term into the model, and if that shows a significant difference, you could stratify by gender and look at the association with the other covariate.

The same can be done for age. Since there are many age groups, you can add one covariate at a time to the same model to see which variable attenuates the odds ratio, and thereby see the effect of the variable. It is believed that presenting such a method would further lead to better discussions and conclusions

Response: Thank you for your valuable feedback. We did not actually build any univariate models and have replaced the term “univariate” in the abstract with “chi-square”. Table 2 consists of proportions and frequencies tested for significance with the chisquare test.

We appreciate your suggestions regarding the analysis methods. While the proposed approaches are indeed insightful and could provide additional depth, they fall outside the scope of our current research question. Our analysis, as submitted, adequately addresses the posed research question. We recognize that exploring these additional methods could constitute a comprehensive analysis deserving of its own dedicated study. We will consider these suggestions for future research endeavors.
